# Questioning Glutamate Excitotoxicity in Acute Brain Damage: The Importance of Spreading Depolarization

**DOI:** 10.1007/s12028-021-01429-4

**Published:** 2022-02-22

**Authors:** R. David Andrew, Eszter Farkas, Jed A. Hartings, K. C. Brennan, Oscar Herreras, Michael Müller, Sergei. A. Kirov, Cenk Ayata, Nikita Ollen-Bittle, Clemens Reiffurth, Omer Revah, R. Meldrum Robertson, Ken D. Dawson-Scully, Ghanim Ullah, Jens P. Dreier

**Affiliations:** 1 Present Address: Queen’s University, Kingston, ON Canada; 2 Hungarian Centre of Excellence for Molecular Medicine-University of Szeged, Cerebral Blood Flow and Metabolism Research Group, Department of Cell Biology and Molecular Medicine, University of Szeged, Szeged, Hungary; 3 University of Cincinnati, Cincinnati, OH USA; 4 The University of Utah, Salt Lake City, UT USA; 5 Instituto de Neurobiologia Ramon y Cajal, Madrid, Spain; 6 University of Göttingen, Göttingen, Germany; 7 Medical College of Georgia, Augusta, GA USA; 8 Harvard Medical School, Harvard University, Boston, MA USA; 9 University of Western Ontario, London, ON Canada; 10 Center for Stroke Research Berlin, Berlin, Germany; 11 Department of Experimental Neurology, Charité – Universitätsmedizin Berlin, Berlin, Germany; 12 School of Medicine, Stanford University, Stanford, CA USA; 13 Florida Atlantic University, Boca Raton, FL USA; 14 University of South Florida, Tampa, FL USA; 15 Department of Neurology, Charité – Universitätsmedizin Berlin, Berlin, Germany; 16 Department of Neurology, Corporate Member of Freie Universität Berlin, Berlin, Germany; 17 Department of Neurology, Humboldt-Universität zu Berlin, Berlin, Germany; 18 Department of Neurology, Berlin Institute of Health, Berlin, Germany; 19 Bernstein Center for Computational Neuroscience Berlin, Berlin, Germany; 20 Einstein Center for Neurosciences Berlin, Berlin, Germany

**Keywords:** Stroke, Traumatic brain injury, Sudden cardiac arrest, Concussion, Modeling, Migraine, Ischemia, Na^+^/K^+^ pump, Huntington disease, Alzheimer disease, Amyotrophic lateral sclerosis, Ketamine, Penumbra, Persistent vegetative state, Dendritic beading, Brain swelling

## Abstract

**Background:**

Within 2 min of severe ischemia, spreading depolarization (SD) propagates like a wave through compromised gray matter of the higher brain. More SDs arise over hours in adjacent tissue, expanding the neuronal damage. This period represents a therapeutic window to inhibit SD and so reduce impending tissue injury. Yet most neuroscientists assume that the course of early brain injury can be explained by glutamate excitotoxicity, the concept that immediate glutamate release promotes early and downstream brain injury. There are many problems with glutamate release being the unseen culprit, the most practical being that the concept has yielded zero therapeutics over the past 30 years. But the basic science is also flawed, arising from dubious foundational observations beginning in the 1950s

**Methods:**

Literature pertaining to excitotoxicity and to SD over the past 60 years is critiqued.

**Results:**

Excitotoxicity theory centers on the immediate and excessive release of glutamate with resulting neuronal hyperexcitation. This instigates poststroke cascades with subsequent secondary neuronal injury. By contrast, SD theory argues that although SD evokes some brief glutamate release, acute neuronal damage and the subsequent cascade of injury to neurons are elicited by the metabolic stress of SD, not by excessive glutamate release. The challenge we present here is to find new clinical targets based on more informed basic science. This is motivated by the continuing failure by neuroscientists and by industry to develop drugs that can reduce brain injury following ischemic stroke, traumatic brain injury, or sudden cardiac arrest. One important step is to recognize that SD plays a central role in promoting early neuronal damage. We argue that uncovering the molecular biology of SD initiation and propagation is essential because ischemic neurons are usually not acutely injured unless SD propagates through them. The role of glutamate excitotoxicity theory and how it has shaped SD research is then addressed, followed by a critique of its fading relevance to the study of brain injury.

**Conclusions:**

Spreading depolarizations better account for the acute neuronal injury arising from brain ischemia than does the early and excessive release of glutamate.

## Introduction

### Spreading Depolarizations and Brain Ischemia

Year after year, published reviews imply that the basic cellular mechanisms underlying acute neuronal death following brain ischemia are reasonably well established based on glutamate excitotoxicity theory. But in fact, the concept that glutamate release by overexcited neurons leads to short- and long-term brain cell death is neither well supported by basic science nor well supported by clinical evidence. In contrast, the role of spreading depolarization (SD) in evoking acute brain damage is more compelling despite SD being underestimated or ignored for years by most researchers of brain ischemia. In this review, we briefly outline the importance of SD in early brain injury, with evidence presented in more detail in the accompanying reviews published in the current issue of Neurocritical Care [1]. We then address the glutamate excitotoxicity theory and compare it with the SD theory in terms of interpreting acute brain injury.

SD is the principal mechanism of electrochemical membrane disruption and neuronal swelling [2–5] in gray matter of the higher brain. Severe ischemia depletes the adenosine triphosphate (ATP) pool, leading to Na^+^/K^+^ pump failure, which generates a front of cellular depolarization that propagates at 1–9 mm/min through the ischemic gray matter and even into healthy tissue [6–8]. The SD wave is a sudden loss of membrane potential to near-zero millivolts that occurs over seconds. SD can be evoked by various noxious electrical, chemical, thermal, or mechanical disturbances of gray matter that stress the Na^+^/K^+^ pump. Thus, SD is associated with a range of diseases and conditions, including migraine-associated aura, concussion, traumatic brain injury (TBI), subarachnoid hemorrhage, intracerebral hemorrhage, ischemic stroke, circulatory arrest, and brain death prior to circulatory collapse [1, 9–14]. SD can also invade gray matter that has not been metabolically compromised, inducing a loss of neuronal activity termed “spreading depression” [15].

In the ischemic core, neurons will die under a maintained depolarization that typically lasts 20–30 min or more [16–19]. However, if the ischemic core is reperfused within ~ 15 min, all neurons will survive, even though persistently depolarized for about 15 min [18, 20]. In contrast, perfusion completely ceases after cardiac arrest, and so neurons start to die after about 5 min [21]. In milder in vivo models, there is no terminal SD (those with no recovery), but typically a cluster of recurrent moderately prolonged SDs occurs superimposed on a relatively shallow negative ultraslow potential. Yet cell death also develops. Clustered SD events pose particularly high metabolic challenge for recovery.

The delayed nature of penumbral SDs presents a potential therapeutic window, whereby their inhibition could improve neurological outcome [22]. These SDs appear to arise as a consequence of energy supply–demand mismatch [23, 24]. The cumulative effect of many secondary SDs is a progressive deterioration of metabolic status and lesion expansion. This occurs because of cytotoxic membrane failure as well as SD evoking microvascular constriction in injured tissue with impaired neurovascular coupling. Known as “spreading ischemia” [25], this inverse hemodynamic or initially vasoconstrictive response to SD promotes prolongation of the cellular depolarization and thus cell death [7, 9, 24, 26–28].

In brain trauma as well as ischemic and hemorrhage stroke, 50–90% of patients exhibit neocortical SDs, and many show continuous, repetitive events lasting several days or even weeks after injury, with total counts of 50 to 100 or more. Even terminal SDs have been observed as the correlate of newly developing focal infarcts and of brain death at end-of-life [10, 11, 18, 29]. The full continuum from the normal hyperemic to the inverse ischemic response to SD has been found in patients with aneurysmal subarachnoid hemorrhage [18, 30, 31], TBI [32], and malignant hemispheric stroke [8].

**Fig. 1:**
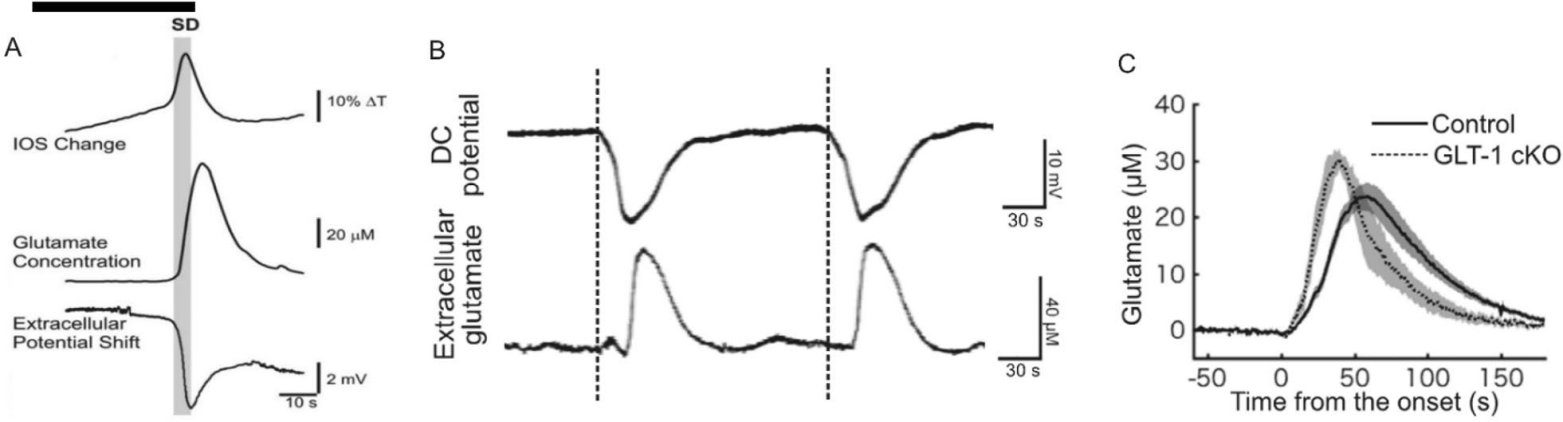
In brain slices, SD is well underway before the extracellular glutamate concentration climbs significantly. **A** The intrinsic optical signal (IOS) change (top trace) was temporally correlated with the DC negative shift (bottom trace). Both signals denote SD onset (shaded region). The glutamate efflux transient is shown in the middle trace. The horizontal bar indicates [K^+^]_o_ elevation to 40 mM for 80 s, thereby inducing SD. From Dr. N. Zhou’s doctoral thesis [161]. **B** More recent recordings using a finer glutamate biosensor also implicated SD as preceding glutamate release. **C** During SD, glutamate release took ~ 35 s to peak. In mice where the glutamate transporter GLT-1 is knocked out, glutamate uptake is slowed. **B** and **C** from [83]; **B** is digitally stretched horizontally from the original. In such multi-recording studies, it is important that sensors are closely placed to precisely determine signal onset times, as further demonstrated in Fig. 2

**Fig. 2:**
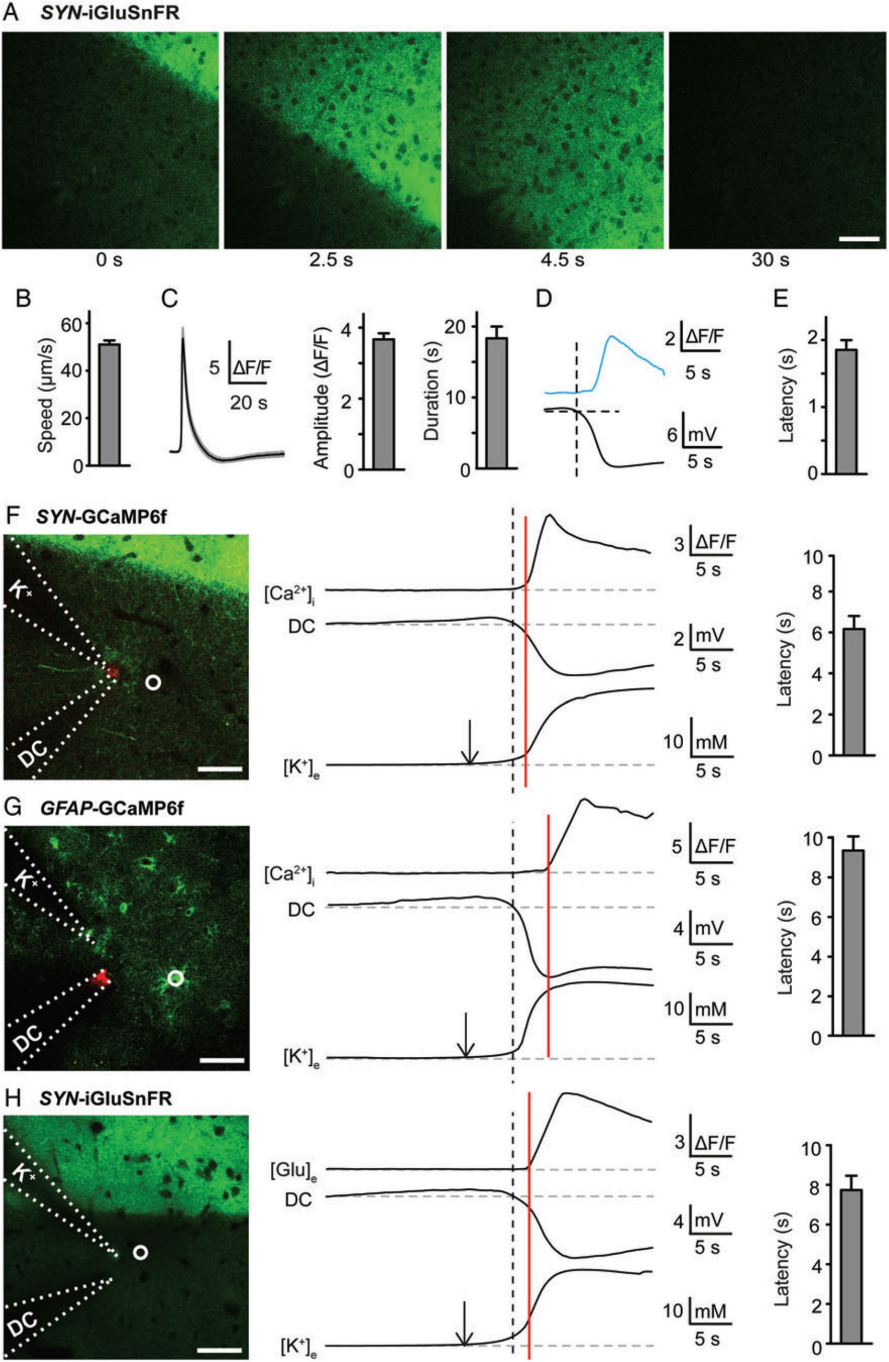
Dynamics of extracellular glutamate and K^+^ during SD in vivo. The SD was evoked at a distance by applying a droplet of 1 mM KCl to the surface of the mouse brain. **A** iGluSnFR, a fluorescent probe, monitors the extracellular glutamate level. **B** Speed of the extracellular glutamate wave. **C** Average fluorescence traces with 95% confidence interval, amplitude, and duration of the glutamate transient. **D** Glutamate trace (blue) aligned to the DC potential below. **E** Latency between negative DC deflection and glutamate increase. **F** Relationship between [K^+^]_o_, DC potential, and neuronal [Ca^2+^]_i_. The latency between 0.25 mM [K^+^]_o_ rise (arrow over K^+^ trace) and increase in fluorescence (red vertical line) is indicated to the right. Dashed line indicates start of the negative DC potential shift. **G** As in **F** but with [Ca^2+^]_i_ in astrocytes instead of neurons. **H** As in **F** but with [glutamate]e instead of neuronal [Ca^2+^]_i_. Images in **F**–**H** show positions of electrodes (stippled lines) and sampled regions (white circles). Sampled regions were picked along the front edge of the SD wave as it hit the K^+^-sensitive microelectrode. Scale bars: 50 μm; error bars, SEM. From [85]

The Na^+^/K^+^ ATPase is the main transporter regulating transmembrane cationic gradients. Its compromise leads to SD. The pump exchanges three cytosolic Na^+^ for two extracellular K^+^ via hydrolysis of ATP [33]. In mammalian gray matter, the Na^+^/K^+^ pump is responsible for ~ 50% of ATP hydrolysis [34]. Lack of blood oxygen and glucose inhibits ATP production, with pump failure evoking sudden SD, driven by the opening of a cryptic Na^+^/K^+^ current [35, 36]. Neurons in live slices undergo SD in response to oxygen–glucose deprivation (OGD) [1, 37–39] (Fig. 1). Similarly SD is imaged and recorded in vivo under ischemia [1, 40–42] (Fig. 2). In both cases, it is the twin stresses of Na^+^/K^+^ pump failure combined with the energy-demanding requirement to repolarize that can kill or injure neurons. Yet the central role of the Na^+^/K^+^ ATPase is rarely considered in explaining excitotoxic mechanisms.

Rather, it is accepted by many researchers that the glutamate excitotoxicity theory essentially accounts for the acute neuronal swelling and brain injury that follows ischemia. Simply stated, on reduced blood flow to the brain, nerve cells suffer overexcitation, swelling, and death when the neurotransmitter glutamate is released or is not retaken up. Pathologically high glutamate levels are proposed to overstimulate its receptors, inducing further excitation in a vicious cycle of release and excitation. This neuronal firing leads to high levels of intraneuronal Ca^2+^, which activates enzymes (phospholipases, endonucleases, and proteases), thereby damaging the cytoskeleton, membrane, and DNA. At least, that is the original textbook story, and numerous modifications have ensued to shore up the concept. However, there are many problems with glutamate being the culprit in brain swelling and neuronal death [4], the most practical being that the concept has yielded zero therapeutics despite decades of work. Moreover, the basic science related to the glutamate excitotoxicity theory must also be reexamined, as well as the assumption that this theory can be extended to yield insights to other central nervous system (CNS) disorders.

The purpose of this critique is to compare SD theory, briefly outlined above and described in detail in an accompanying review [1], with glutamate excitotoxicity theory. Additionally, we explore whether these two concepts might have some common ground. We start with briefly examining the historical basis for the acceptance of elevated glutamate levels being the trigger for activating a cascade of biochemical events that kills neurons.

### How did Excessive Glutamate Release Gain Traction as a Major Cause of Brain Injury?

Glutamate release caused by ischemia has been de facto accepted as the cause of stroke injury for more than 35 years. This developed from a series of four foundational observations beginning in the 1950s. The first finding was that excess glutamate injection injured the mouse CNS. But to produce a severe retinal lesion, Lucas and Newhouse [43] in 1957 showed that a parenteral dosage of “a little less than lethal was needed.” In 1969 Olney [44] administered a near-lethal single dose of glutamate subcutaneously, which caused massive brain lesions, later showing that glutamate analogues at high concentration were also effective. A second observation was that ischemia caused glutamate release, an unsurprising finding given that neurons depolarize and that about 70% of CNS neurons are glutamatergic. It is important to note that the extracellular increase was short lived and included other neurotransmitters [45, 46]. For example, OGD-evoked dopamine release is particularly high in striatal slices [47], as is gamma aminobutryic acid (GABA) release in hippocampal slices [48]. The third finding by Choi et al. [49] in 1987 was that glutamate receptor (gluR) antagonists protected cultured neurons from glutamate toxicity. However, note that cultured neurons, being derived from immature tissue and having adapted to living in a dish, can tolerate long periods of OGD and do not undergo the incisive event of early stroke ischemic SD. The fourth observation was that *N*-methyl-d-aspartate receptor (NMDAR) antagonists were reported to reduce ischemia-induced neuronal damage in rodents in vivo. But hundreds of compounds unrelated to gluRs showed efficacy, most reported in rodent models of ischemia over the past 30 years [50]. Yet no drug has proven to be protective in clinical trials of ischemia. Twenty-eight suspected inhibitors of excitotoxicity were tested preclinically in patients with acute stroke in 1993–2001 [50], but none proved to be neuroprotective. Two other potentially useful drugs, ketamine (“Ketamine and SD Inhibition” section) and NA-1 (“Therapeutic Hope for Combating Glutamate Excitotoxicity?” section), antagonize NMDARs but may also have broader actions.

The many rationalizations for these negative results have included claims of too broad patient selection criteria, problems with delayed intervention, poor tolerability by patients, publication bias in favor of positive results (perhaps for commercial or other reasons), quality of the molecules (pharmacokinetic deficiencies), inability to reach effective concentrations in the penumbra, inappropriate neuroprotective time window, insufficient receptor subunit selectivity, high drug toxicity in humans, inequivalent doses compared to rodents, development of tolerance (eg, upregulation of NMDARs), and finally side effects blocking normal synaptic *N*-methyl-d-aspartate (NMDA) activity that would have promoted neuronal survival [51]. Some of our authors feel that it is still worthwhile to search for clinical applications for gluR antagonists. Nevertheless, it is surprising that this litany of excuses has rarely led to questioning the basic science that has portrayed glutamate release as the great destroyer of the CNS. A handful of publications provide exceptions [52–55].

## Glutamate Release and SD

### Use of Antagonists to Block SD in Live Brain Slices

Glutamate was originally considered a contender as an SD mediator because of its progressive release into the interstitial space during brain ischemia [56]. There is a long history of studies using live brain slices with the goal of identifying how SD is initiated and sustained. A common presumption has been that glutamate release must have a central role. Various inhibitors of channel opening, of transmitter receptors, or of transporters have been bath-applied to test which mechanisms elicit SD (Table 1).

**Table 1:** Studies using live brain slices to test one or more drugs that might block SD under the energy compromise imparted by hypoxia or OGD

Study	Stimulus	Glutamate receptor blockers	Na^+^ channel blockers	GABA_A_ receptor blockers	Ca^2+^ channel blockers	Other channels blocked	Recording mode	SD blocked? (in some slices)	SD imaged?
Radek and Giardina [75]	Hypoxia	100 µM AP-5						Yes	No
Tanaka et al. [39]-drugs tested separately	OGD	50–250 µM AP-5 or 10–20 µM CNQX		20 µM bicuculline			Intracellular	No	No
Yamamoto et al. [64]-drugs tested separately	OGD	50–250 µM AP-5 or 10–20 µM CNQX	Procaine (0.3–1 mM) or TTX (0.3 μM)		2 mM Co^2+^ 2 mM Ni^2+^ or 10 μM nifedipine	K^+^: (20 mM TEA)	Intracellular	No	No
Müller and Somjen [70]	Hypoxia	10 µM CPP or 10 µM DNQX	1 µM TTX		2 mM Ni^2+^		Field recording or imaging	Partly	Yes
Müller and Somjen [164]	Hypoxia	10 µM CPP or 10 µM DNQX	1 µM TTX				Field recording or intracellular	No in 50% of neurons	No
Rossi et al. [88]	Chemical ischemia + OGD	50 µM D-AP5, 50 µM MK-801, 25 µM NBQX, 100 µM 7-chlorokynurenate		100 µM bicuculline			Intracellular	No, but yes in a few slices when glutamate transport also inhibited	No
Jarvis et al. [163] (rat) Joshi and Andrew [38] (mouse)	OGD	50 μM AP-5 + 10 μM CNQX or 2 mM kynurenate					Field recording or intracellular	Delayed	Yes
Anderson et al. [37]	OGD					Sigma receptor-activated	Imaging	Yes	Yes
Madry et al. [162]	Chemical ischemia + OGD	50 µM D-AP5, 50 µM MK-801, 25 µM NBQX, 100 µM 7-chlorokynurenate		100 µM bicuculline		Pannexin block had no effect	Intracellular	Yes, but residual current	No
Douglas et al. [57]	OGD		1–10 µM dibucaine or other caines				Imaging	Yes	Yes
Revah et al. [71]	Hypoxia	50 μM APV, 50 μM MK-801, 50 μM DNQX, 25 µM NBQX		25 μM bicuculline		Pannexin block had no effect	Intracellular	Yes	No
Gagolewicz et al. [165]	OGD	1 mM kynurenic acid	1 μM TTX	100 μM picrotoxin	10 μM nifedipine	K^+^: (10 mM TEA); pannexin block had no effect	Intracellular	No	Yes
Gagolewicz et al. [165]	OGD					Block of ASIC, P27X, and TRPM7 channels + glutamate transport inhibition had no effect	Intracellular	No	Yes

GluR antagonists, applied either separately or as a cocktail, do not block SD in the majority of studies of OGD. Being less metabolically demanding, hypoxic SD is more easily inhibited. Where SD is reported as blocked, a caveat is that SD may have occurred outside the recorded cell’s area, unless confirmed with imaging. Invariably, none of these studies block SD in all slices tested

*N*-methyl-d-aspartate (NMDA) receptor antagonists inhibit NMDA receptors: *AP5 or APV* DL-2-amino-5-phosphono-valerate, *CPP* (±)-3-(2-carboxypiperazin-4-yl)-propyl-1-phosphonic acid, *ketamine* 2-(2-chlorophenyl)-2-(methylamino)cyclohexan-1-one,* MK-801* (5R,10S)-(+)-5-methyl-10,11-dihydro-5H-dibenzo[a,d]cyclohepten-5,10-imine

Non-NMDA receptor antagonists inhibit AMPA/kainate receptors: *CNQX* 6-cyano-7-nitroquinoxaline2,3-dione, *DNQX* 6,7-dinitroquinoxaline-2,3-dione, *NBQX* 2,3-dioxo-6-nitro-1,2,3,4-tetrahydrobenzo[f]quinoxaline-7-sulfonamide. *TEA* tetraethylammonium, *TTX* tetrodotoxin. For additional treatments that do not block OGD–SD, see [63, 64]

*Kynurenate* 3-Anthraniloyl-l-alanine, broadly inhibits glutamate receptors

^1^Some sigma receptor ligands dramatically inhibit SD onset [37], but the function of sigma receptors remains unclear

^2^Two studies, [57, 58], showed neuronal protection from SD initiation and injury by 1–10 mM dibucaine, the most potent Na^+^ channel blocker of the caine group

^3^OGD-induced intracellular Ca^2+^ increases are mediated by Ca^2+^ influx through NMDARs, VGCCs and TRPC channels as well as by Ca^2+^ release from RyRs and IP3Rs. This impairs mitochondria, facilitating SD generation [63]

#### Na^+^ Channel Blockers

**Fig. 3:**
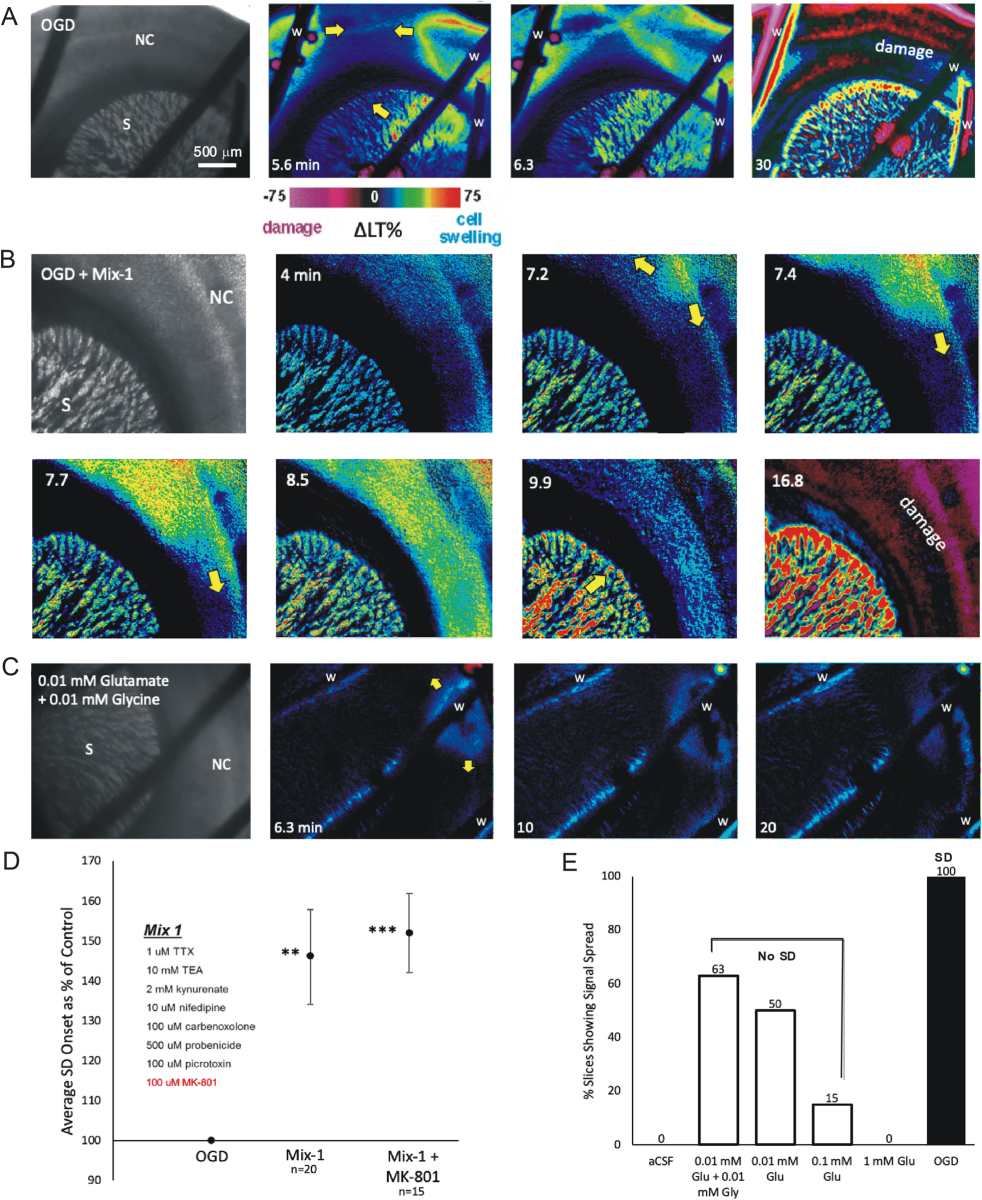
Unlike O_2_/glucose deprivation (OGD), bath superfusion of glutamate at pathophysiological concentrations onto a brain slice does not induce SD. **A** Imaging change in light transmittance (ΔLT) reveals OGD-induced SD and propagation (arrows) along neocortical gray matter (NC) and through striatum (S) with damage arising in the wake of SD (magenta). w = slice weight. **B** A cocktail of blockers (Mix-1, constituents listed in Fig. **D**) delays OGD-induced SD onset but not propagation (arrows). By 16.8 min, light scattering in NC caused by dendritic beading indicates acute neuronal damage. **C** Bath superfusion of glutamate causes slight signal creep from the overlying weight, but no SD. **D** Superfusion of Mix-1 significantly (*p* = 0.002) delayed OGD-induced SD onset by 46 ± 11.8%. Adding MK-801 slightly but significantly (*p* < 0.0001) further delayed SD onset by 52 ± 9.9%. **E** Percent of slices generating signal creep but no SD in three experimental groups of glutamate application to naïve slices. No SD was observed in slices superfused in aCSF alone (*n* = 6) or in aCSF + 1 mM glutamate (*n* = 6)

The Na^+^ channel blocker tetrodotoxin (which silences all action potential activity) does not block either hypoxia- or OGD-induced SD (Fig. 3b, d), indicating that spike-activated synaptic release of neurotransmitters is not a requirement for SD. Likewise, Na^+^ channel blockers composing the caine family (dibucaine, lidocaine, procaine, etc.) silence action potential firing but merely delay OGD–SD onset [46, 57–59]. And in vivo, lidocaine only delays SD and is not neuroprotective [21]. Although not necessary for SD generation, V-sensitive Na^+^ channel opening supports SD onset [60].

#### NMDAR Antagonists

NMDAR antagonists tested individually could indeed inhibit the propagation of SD in nonmetabolically stressed brain slices [61]. Under hypoxia in slices, NMDAR antagonists generally delay but cannot block SD [62]; however two hypoxia studies did report blockade (Table 1), so the results are inconsistent. NMDAR antagonists did not prevent SD evoked by lowering both oxygen and glucose levels [39, 63, 64].

#### Non-NMDAR Antagonists

Tested individually, these inhibitors of α-amino-3-hydroxy-5-methyl-4-isoxazolepropionic acid (AMPA) and kynurenate receptor binding could delay but, again, not block OGD-evoked SD [39, 64] (Table 1).

#### Antagonist Cocktails

Slice researchers have also combined blockers (Table 1). Early slice studies showed that gluR antagonists can delay or stop the onset of hypoxic SD [62] but only delay SD evoked by OGD [36, 39, 64, 65], findings supported in many but not all subsequent slice studies (Table 1, Fig. 3b, d). Commonly the cocktails include antagonists of gluRs, of glutamate transport, and/or of cation channels to isolate conductances required for SD generation and propagation. There are variations in cocktail constituents, in the mode of SD induction, and in the degree of SD inhibition. Additionally, there is no reported drug reversibility because of high antagonist concentration, tight chemical binding, or both.

The role of gluRs in SD generation remains a contentious issue for several reasons. Normoxic and hypoxic SDs are more easily inhibited than ischemic SD both in vivo and in slices. Finally, note in Table 1 that cocktails may contain one or more non-NMDAR antagonists (CNQX (6-cyano-7-nitroquinoxaline2,3-dione), DNQX (6,7-dinitroquinoxaline-2,3-dione), NBQX (2,3-dioxo-6-nitro-1,2,3,4-tetrahydrobenzo[f]quinoxaline-7-sulfonamide)) at concentrations between 25 and 100 μM. Yet these drugs are approximately equipotent at the same AMPA receptors and are fully potent in hippocampal slices at only 3 to 5 μM based on several standard electrophysiological paradigms [66]. Likewise, the NMDAR antagonist MK-801 applied at 50 μM nevertheless fully blocks evoked field potentials in slices at 1 to 2 μM [67, 68]. This is also an issue with the wide-spectrum gluR antagonist, kynurenate. It selectively blocks responses to NMDA at 200 μM [69] yet is incapable of blocking OGD–SD at 1 to 2 mM (Fig. 3b, d, Table 1). So gluR antagonists are commonly bath-applied at much higher concentrations than the effective dose required to block the receptors during normal synaptic transmission.

These gluR blocker levels may represent pharmacological overkill. This is an important point because Tanaka et al. [39] showed that individually increasing AP-5 stepwise from 50 to 100 μM, and then to 200 μM, significantly delayed OGD–SD onset longer at each step. Likewise, individually increasing CNQX from 10 to 20 μM significantly delayed OGD–SD onset further. This suggests that including enough gluR blockers in a cocktail at concentrations that exceed their effective physiological thresholds for synaptic blockade can cause cumulative and nonspecific effects. Most importantly, this includes a nonreversible silencing of the gray matter, making it appear that SD has been blocked [70]. This issue of the potentially toxic effects by gluR antagonists was discussed by Revah et al. [71]. They noted that the high levels used in slice studies had confounded their use clinically. Whether lower levels of gluR antagonists might prove therapeutically useful is worth pursuing.

#### False SD Blockade

Another reason why potential SD inhibitors may appear to block SD (but do not) is the issue of “false positives in SD suppression” [72]. When SD is imaged in vivo [73] or in slices, SD often does not invade the entire field under view. If a neuron is recorded without imaging of the entire slice, it is uncertain if the SD front has actually reached the recorded cell. If not, SD would appear to be blocked. Likewise, the lack of a negative direct current (DC) shift recorded near the recorded neuron may represent a true blockade of SD. Alternately, SD simply did not invade the locale of the field recording. Therefore, in studies in which complete SD blockade is reported (Table 1), either imaging or multiple electrode recordings would confirm that SD was indeed blocked throughout a recorded slice.

Despite variation in slice study findings, several points of consensus stand out:

1. Inhibiting OGD-induced SD is difficult; hence, an unidentified channel (or channels) responsible for generating SD is likely. One proposal being pursued is that the Na^+^/K^+^ pump converts to an open channel [74].
2. The opening of standard Na^+^, Ca^2+^, or K^+^ channels is not necessary for SD generation.
3. High concentrations of toxic drugs are less effective in inhibiting SD than simply lowering slice temperature, which protects neurons from hypoxia [75] or OGD [38].

### Nonsynaptic release of extracellular glutamate during SD

In hippocampal slices, the orthodromically evoked CA1 field potential is typically lost prior to hypoxia- and OGD-induced SD onset [76] (Fig. 10c in Ref. [37]). The effect is less obvious in neocortical slices, where SD onset has a shorter latency (Fig. 11b in Ref. [37]). The loss of the synaptic response in CA1 prior to SD may be an early effect of lowered ATP levels because it is not seen prior to SD evoked by ouabain or high K^+^ levels, in which ATP production is not compromised as with OGD. Additionally, an early release of the presynaptic blocker adenosine has also been proposed [77]. However, the OGD-evoked loss of the synaptic response raises the question as to how the extracellular glutamate concentration ([glu]_o_) begins to climb because of SD onset. There are several proposed mechanisms to account for this increase, including (1) reversal of glutamate transporters [78], (2) release by volume-activated channels in astrocytes [79], (3) release via presynaptic NMDAR activation [80], (4) release of a small but readily releasable pool (RRP) of glutamate [71, 81], (5) Ca^2+^-dependent vesicular glutamate release from astrocytes (although this process has not been directly linked to SD), and (6) a major source of extrasynaptic glutamate (the cystine/glutamate antiporter) stimulated by ischemia [82]. Specifically, Revah et al. [71] showed that hypoxia can elicit a small RRP of glutamate from axon terminals in slices. The RRP is not linked to action potential invasion of the terminal, which means that there is then less available glutamate for regular synaptic release. This could potentially contribute to the eventual synaptic failure that precedes SD initiation under hypoxia or OGD. However, there are, as of yet, no recording techniques sensitive enough to directly detect the small [glu]_o_ increase originating from the RRP. So there are several sources of extracellular glutamate that could support SD ignition evoked either by elevated extracellular potassium concentration ([K^+^]_o_) levels or by hypoxia. Nonetheless, with the greater metabolic load of OGD, SD persists in the presence of gluR antagonists in most but not all studies, both in vivo and in acute slices (“Use of Antagonists to Block SD in Live Brain Slices” section).

Importantly, there is no evidence to date that the initiation of any form of SD onset is preceded by measurable glutamate release. Early in vivo studies showed that a [glu]_o_ increase followed local OGD–SD onset, as measured by the initial DC shift or by the slight upswing in [K^+^]_o_ [55]. A glutamate surge was also detected in slices within seconds of the SD front passing [83, 84] (Fig. 1a). More recently, use of genetically encoded optical glutamate sensors allowed improved spatiotemporal resolution of transient glutamate levels near the SD front measured as the negative DC shift [85]. The onset of the DC shift coincided with the first increases in [K^+^]_o_, but coregional [glu]_o_ elevation began only after the DC shift, and [K^+^]_o_ had been increasing for 2–3 s (Fig. 1b, c). Thus, elevated [glu]_o_ in the locality of SD initiation appears to be a result, rather than a cause, of ischemic or hypoxic SD. This is not to say that elevated [glu]_o_ cannot increase SD propensity in normoxic situations [86, 87].

### Glutamate Transporter Studies and OGD-Induced SD

Reduced reuptake of glutamate as a promoter of ischemia-like SD was examined by Rossi et al. [88] using hippocampal slices. Although there are no specific blockers of glutamate transporters, pretreatment with the glutamate analogue l-trans-pyrrolidine-2,4-dicarboxylic acid (PDC) slows glutamate release via reverse transport induced by simulated ischemia. Their finding that PDC pretreatment blocked ischemic SD led them to conclude that the increased [glu]_o_ caused by reversed glutamate transport plays a key role in generating SD. However, SD was blocked in only 4 of their 19 slices. In five other slices, the SD was smaller and slower in onset. Full-blown SD was still generated in their remaining ten slices. In the five slices in which SD appeared to be blocked, there remained the possibility that SD was generated and propagated elsewhere in the slice but might not have invaded the locale of the recording (Table 1). So, although reversed glutamate transport may contribute to elevated [glu]_o_ during ischemia, the argument that it plays a key role in SD generation remains unconvincing. Also, transporter reversal by ischemia requires the rundown of ion gradients, which is the result of SD, not the cause. That is, SD precedes the reversal of glutamate transport. Moreover, an earlier in vivo study found that PDC microdialysis hastened SD onset in the neocortex but not in the hippocampus and striatum, despite extracellular glutamate being markedly increased by PDC [89].

A recent study showed that inhibiting one particular excitatory amino acid pump (Glu-1, i.e., GLT-1) but not two other glutamate transporters promoted nonischemic SD [83]. Again, however, glutamate release lagged SD initiation (Fig. 1b). Another study showed that although the antibiotic ceftriaxone increased expression of GLT-1 and lowered extracellular glutamate, it did not alter SD frequency (also induced by KCl injection) [90]. Thus, there has been no consistent promotion of SD reported by reversing glutamate uptake.

### Bath Application of Glutamate in Brain Slices

Bath exposure to glutamate (up to 0.1 mM, which is pathophysiological) induces depolarization and a negative DC shift wherever one chooses to record in a slice. Imaging light transmittance changes in an entire slice shows that this depolarization arises in the majority of imaged gray matter almost simultaneously, i.e., the depolarization does not spread throughout neocortical and hippocampal gray matter. A range of concentrations of bath-applied glutamate (0.01–0.10 mM; Fig. 3c, e) fails to induce an SD [91–94]. So the elevated light transmittance signal evoked by glutamate (denoting neuronal swelling and depolarization [95] develops uniformly in the slice, does not propagate, and is blocked by AP-5. This contrasts with bath exposure to ouabain, palytoxin, OGD, and hypoxia, all of which inhibit the Na^+^/K^+^ pump and so reliably elicit SD. So pump compromise, rather than rising glutamate levels, leads to SD. Depolarization caused by SD involves opening of a Na^+^/K^+^ conductance, whereas glutamate depolarizes by activating gluRs, a completely different mechanism.

A stimulus causing depolarization of hundreds of neurons in a confined space (e.g., focal application of KCl or pin prick) will increase local [K^+^]_o_, thereby setting off an SD wave independent of gluR activation. However, because gray matter remote from the injury is not stressed, gluR antagonists can inhibit SD propagation there [61, 96, 97]. Other examples of focal stress evoking SD in slices are the dropping of a small weight on a slice [98], KCl ejection [99–101], and 0.1-mM application of NMDA [80]. Therefore, the original report that local application of high concentrations of glutamate to the cortical surface [56] is not, in and of itself, convincing evidence that an elevated extracellular glutamate level is the activator of SD. But it can support SD propagation through less metabolically stressed gray matter.

### Ketamine and SD Inhibition

Ketamine has gained increasing attention as a therapeutic [102]. Retrospectively, it was found to cause a dose-dependent reduction in SD events in a mixed, but small, population of patients with TBI, aneurysmal subarachnoid hemorrhage, or malignant hemispheric stroke [103, 104]. The first prospective controlled clinical trial testing ketamine confirmed this in eight patients with TBI and two patients with subarachnoid hemorrhage [29]. In this study, SD inhibition was observed near the region of injury, indicating that inhibition was not restricted to the healthier portion of the injury gradient. In a recent case series of 66 patients with subarachnoid hemorrhage, low- and high-dose administration of s-ketamine was retrospectively compared [105]. The high-dose range was above the upper limit for sedation recommended by the manufacturer but resulted in further significant decrease in SD incidence. A similar dose–response relationship was also seen in swine in vivo. In these animals, SDs were triggered with 1-µL drops of 1 M KCl after preconditioning the cortex with Ringer’s solution containing KCl at 11 mM for 1 h and covering it with paraffin thereafter. s-Ketamine at 2 mg per kg of body weight per hour then decreased the expansion, amplitude, duration and speed of SDs but only completely blocked them in this well perfused, almost normal tissue at a high dose of 4 mg per kg of body weight per hour applied over a time period of 2 h [106]. This suggested that the assumed neuroprotective effect of s-ketamine in the swine essentially starts at a range tolerated by neurointensivists in humans in individual cases but above the recommended one, so a concern is pharmacological side effects. For example, ketamine is not only an NMDAR inhibitor but also inhibits norepinephrine and serotonin uptake, which could damage, for example, the kidneys. Yet in the clinical study [105], there was no evidence of acute kidney injury [107]. This could be because patients with brain injury are usually treated with high doses of catecholamines and that ketamine reduces the required dose because it inhibits norepinephrine uptake [108].

Reinhart and Shuttleworth [109] showed in a brain slice model, up to 30 μM ketamine did not block K^+^-induced SD but reduced its duration. In slices under partial metabolic compromise (artificial cerebral spinal fluid (aCSF) flow restricted to one side of the slice), low-dose ketamine again did not inhibit K^+^-induced SD but helped protect against resulting neuronal injury in the metabolically stressed gray matter. In another recent study of photothrombosis in rats, electrographic seizure induction by 4-aminopyridine caused no increase in lesion volume or neuronal injury in urethane anesthetized animals. By contrast, ketamine anesthesia was associated with a longer duration of 4-aminopyridine-induced seizures, as it is a weaker antiepileptic drug than urethane. However it lowered SD frequency and reduced both the magnetic-resonance-imaging-based lesion volume and neuronal injury as determined stereotactically [110]. So there is preliminary evidence that ketamine treatment can suppress a fraction of SD events in patients with brain injury and brain-injured animals. Similar to its relative MK-801, it shows neuroprotective effects but at relatively high doses [111–113]. Most likely its neuroprotective effects are due to beneficial effects on tissue that is at risk, but less energy compromised, than tissue in slice models of hypoxia or OGD [114, 115].

### gluR-Targeted Drugs Tested to Protect from SD

There is consensus that SD displays a higher resistance to drugs targeting gluRs as gray matter becomes increasingly energy-depleted [6, 97]. In brain slices or in vivo, gluR antagonists inhibit normoxic and normoglycemic SD in otherwise naïve tissue [61, 116, 117]. In contrast, many slice studies (Table 1) and in vivo studies [118, 119] show that gluR antagonism is not sufficient to block SD in more severely hypoxic or ischemic tissue. Interestingly, NMDAR antagonists also increasingly lose their efficacy to inhibit SDs under increasing baseline [K^+^]_o_ [41]. The latter seemingly contradicts the notion in many reviews that KCl-triggered SD is blocked by NMDAR antagonists. However, the fact is that gluR antagonists only inhibit invasion of SD into normal tissue surrounding a focus of KCl application, similar to their block surrounding an ischemic focus or a point of mechanical/electrical trauma. gluR Antagonists do not necessarily block the ignition of SD at the site of the noxious trigger [3, 6]. NMDAR inhibitors appear to shorten SD events in the normal tissue, until they eventually fail to propagate [96].

So whether brain-injured patients treated with ketamine (instead of other sedatives such as propofol or midazolam) would display fewer SD events with a better clinical outcome remains to be confirmed by controlled clinical trials. In particular, disorders with large brain areas under mild energy deficiency with patients requiring sedation should be studied. Subarachnoid hemorrhage [9], and TBI are promising candidates, especially if there is a pronounced subarachnoid blood component [120]. Such studies should exploit the options offered by modern neuromonitoring technologies. First however, feasibility studies need to examine which criteria should guide the administration of ketamine or alternative sedatives, given that sedation is itself an adverse event that should be avoided when there is no need [16, 31, 120, 121]. Fortunately, there is already extensive clinical experience with ketamine as a sedative. What renders studies with ketamine difficult, however, is its multiple effects, which have earned it the reputation of being “a pharmacologist’s nightmare.” Thus, ketamine is well documented as binding the dizocilpine site of the NMDAR and indeed, the site has a 15-fold selectivity over other sites (https://en.wikipedia.org/wiki/Ketamine). Yet there are substantial interactions with D_2_ receptors, with nicotinic acetylcholine receptors (by ketamine metabolites), and with α estrogen receptors. As well, there are weaker interactions with approximately 20 other neurotransmitter receptors and transporters.

## SD and the Concept of Glutamate Excitotoxicity

Because glutamate excitotoxicity has been considered the dominant trigger of cellular brain injury for 30 + years, most researchers studying SD and stroke assumed a tight relationship between SD, glutamate release, and acute neuronal injury. But for every early SD study supporting the role for glutamate as an excitotoxin, there was another study casting doubt. This prompted some modest re-evaluation and moderation [53, 122, 123] even as doubt continued to mount.

### Intact Rodent Studies Addressing SD and Glutamate Excitotoxicity

NMDAR blockers affected SD waves in a more complex way in vivo than reported in slices. Waves continued to arise and propagate from the vicinity of the K^+^ probe but were very brief in duration [124]. Those waves reaching the NMDA-blocked area could become full-blown, propagating SD waves. Also, SD waves initiated from this region could propagate, albeit with reduced duration. These results proved that glutamate is not essential for SD ignition nor propagation, although it supports the latter. Also, whereas sustained elevation of K^+^ in vitro (i.e., core conditions) produced drastic loss of neuron viability that was only partially recovered by NMDAR blockade [125], the slow mild delivery of K^+^ setting the penumbra-like SD focus in vivo produced lasting damage to dendritic electrogenesis that prevented orthodromic transmission, but it was insufficient to kill neurons [124]. These observations suggested that neither elevated K^+^ nor glutamate alone were sufficient to kill neurons in the ischemic core. Herreras’ group also identified glial disruption of metabolic support to neurons as a harmful element [23].

In vivo data published in the late 1990s by Obrenovitch and colleagues confirmed and extended these observations. They argued that elevated glutamate levels are a result, not a cause, of ischemic SD onset. Using enzyme-amperometry to analyze dialysate glutamate with a high time resolution, they never found any sign of a progressive increase of [glu]_o_ preceding SD under ischemia. This work further dissented from prevailing excitotoxicity theory which claimed that elevated glutamate release was the primary cause of acute ischemic brain injury in stroke [55, 89, 126]. Their work also supplied evidence against glutamate excitotoxicity being a major factor in TBI [122], arguing that gluR blockade simply slows depolarization, thereby lowering the metabolic energy requirements for recovery. Thus, an elevated glutamate level is just one of several contributing effects to ischemia-induced neuronal injury. It has been repeatedly argued that the glutamate hypothesis of excitotoxicity represented an overinterpretation of data from cultured neurons, which was hastily applied to the intact animal [53, 122, 123].

Murphy et al. [40] imaged changes in light reflectance and calcium fluorescence in the intact mouse neocortex to both confirm ischemic SD onset and to delineate the regions that SD invaded following focal ischemia in vivo. The SD wave front and associated dendritic beading were unaffected by pretreatment with either an NMDAR or an AMPA/kainate receptor antagonist. They also found “glutamate receptor-independent ischemic depolarization as the major ionic event associated with disruption of synaptic structure during the first few minutes of ischemia in vivo.” Hinzman et al. [127] stressed that excitotoxicity in acute lesion development occurs as a consequence of SD. They found that [glu]_o_ increased only after MCA occlusion and only in association with SD. It did not increase before SD or independent of SD, either involving the initial SD in the core, or the prolonged SDs in the penumbra.

### Studies Addressing Elevated [Ca^2+^], Ischemia, and Excitotoxicity

Because glutamate activated Ca^2+^ release (both intra-and extracellularly) is a crucial aspect of excitotoxicity theory, there is the question of whether Ca^2+^ release influences SD in ischemic gray matter. In a rat brain slice model, SD was triggered by a rise in K^+^ to 40 mM in the perfusing aCSF over 90 and 120 s [84]. The study indicated that glutamate is released by vesicular exocytosis at the SD front, activating NMDA receptors with consequent indirect Ca^2+^ release from mitochondria in addition to direct Ca^2+^ influx from the extracellular space, followed by glutamate exocytosis at neighboring neurons [84]. However in naïve tissue, indirect Ca^2+^ release from mitochondria seems insufficient to drive this process as, in contrast to the slices exposed to brief K^+^ challenge, removal of extracellular Ca^2+^ or inhibition of voltage-gated Ca^2+^ channels blocks normoxic SD [128, 129]. Moreover, it was independently observed by different research groups that the fall in [Ca^2+^]_o_ follows the neuronal depolarization and increase in [K^+^]_o_ by a few seconds [96, 130–132] (Fig. 2f, g). Toyoda et al. [63] showed that OGD-induced [Ca^2+^]_i_ increases are mediated by Ca^2+^ influx through NMDARs, Voltage-gated calcium channels (VGCCs) and transient receptor potential (TRP) C channels as well as by intracellular Ca^2+^ release. All of this Ca^2+^ movement impairs mitochondria but only slightly facilitates SD generation. It seems likely that the elevated intracellular Ca^2+^ evoked by SD generation is at least as responsible for initiating downstream injury cascades as the glutamate that SD releases.

### Glutamate, Nitrous Oxide, and Reactive Oxygen Species

The extracellular glutamate concentration ([glu]_o_) increases to about 40–50 µM during SD in brain slices [83, 84] (Fig. 1). However, this is insufficient to initiate SD in neighboring tissue as SD induction by brain topical glutamate application in vivo required a concentration of ~ 15 mM [56, 133]. Cortical injection of 1 mM glutamate did not induce SD in vivo, although this resulted in a microelectrode-recorded increase in [glu]_o_ to ~ 250 µM [127]. In addition, glutamate uptake inhibitors can markedly increase [glu]_o_, but they do not induce SD [89, 127] (“Glutamate Transporter Studies and OGD-Induced SD” section). Bath application of Na^+^/K^+^ pump blockers such as ouabain or palytoxin induce SD whereas flooding slices with glutamate or gluR agonists such as NMDA or kainate produces diffuse (non-spreading) depolarization and neuronal swelling rather than the propagating sequence of events typical of SD [91–93]. The diffuse response requires 1 mM glutamate in the bath, which is 50 times the peak [glu]_o_ recorded during SD. In particular, NMDA application onto slices has been represented as a proxy for ischemia [95]. However, as detailed in the next section, NMDA is a synthetic compound, so its effects are not neutralized by glutamate reuptake mechanisms. And as detailed below, NMDAR activation is also not required to induce acute neuronal damage evoked by ischemia in vivo.

Why does flooding the slice with bath-applied glutamate (0.01 to 0.1 mM; Fig. 3c, e), or NMDA or kainate fail to induce an organized spreading event whereas elevating [K^+^]_o_ induces spread? Both released K^+^ or released glutamate will initially be buffered by glial reuptake mechanisms that will be quickly swamped, leading to depolarization of the entire field of neurons. Yet an SD is evoked by bath-applied K^+^ but not bath-applied glutamate. One explanation is that elevated [K^+^]_o_ directly decreases pump activity, whereas massive gluR activation simply depolarizes every neuron at once, later leading to pump overload. We have hypothesized that pump compromise is prerequisite for generating a propagating event. Clearly, the way to fully refute K^+^ or glutamate as the natural driver of SD is to chemically identify a factor that (1) is released by gray matter in response to metabolic stress, (2) becomes elevated just prior to SD onset, and (3) evokes SD.

Glutamate application at 100 µM to primary cell cultures should not be considered a proxy for ischemia, OGD, or hypoxia e.g., [134, 135]. A problem is that molecular oxygen is required for ATP production but also for nitrous oxide (NO) synthesis [136, 137]. So increased [glu]_o_ cannot increase NO production without molecular oxygen. Yet numerous neuroscience textbooks state that ischemic cell damage is a consequence of the excitotoxic increase in NO production caused by glutamate release. Likewise the production of reactive oxygen species (ROS) requires oxygen [138]. Thus, SD-induced glutamate increases under ischemic conditions in vivo cannot cause an increase in ROS production without the return of oxygen, despite glutamate being a potent stimulus of ROS production in cell culture [139]. Elevated ROS production only occurs in the context of ischemia in vivo if the tissue is reperfused, allowing glutamate reuptake [25, 138]. Numerous studies on thrombolysis and mechanical recanalization of stroke patients have now shown that reperfusion does not lead to additional damage, but currently represents the only chance for ischemic brain tissue to survive [140]. So it is debatable whether glutamate release itself increases the NO and ROS that leads to ischemic damage to neurons.

### Glutamate and Nonstroke Neurological Disorders

Most neuroscience textbooks include a diagram outlining how ischemia leads to excess glutamate release, triggering high calcium influx that leads to elevated second messenger systems, thereby mediating neuronal death in various ways. This ongoing scenario is a direct result of the unchallenged foundational studies described in “How did Excessive Glutamate Release Gain Traction as a Major Cause of Brain Injury?” section. And so we ask, Has glutamate excitotoxicity theory in any way contributed to the understanding of dysfunctional brain states beyond ischemia?

A critical review in 2015 [141] concluded that regarding Huntington disease (HD), Alzheimer disease (AD), and amyotrophic lateral sclerosis (ALS), glutamatergic dysregulation might somehow contribute to the varied disease pathology. But the review also noted that the molecular basis of each disorder is multilayered (particularly in ALS and AD) with a very complex profile for each. Importantly, there is no consistent evidence that extracellular cerebral glutamate levels are grossly increased, even in HD where excitotoxicity theory predominated for over 20 years. Lewerenz and Maher [141] conclude “it not clear that therapeutic interventions that re-establish glutamatergic homeostasis during ongoing neurodegeneration will be effective tools for stopping the disease process”. Moreover, glutamatergic dysregulation is undoubtedly not the only process leading to the varied neuronal neurodegeneration of AD, HD, and ALS. Yet many scholarly reviews have titles that impressively imply glutamatergic dysregulation as the clear target for some eventual clinical treatment. A few recent reviews discuss targeting specific gluR subunits but also emphasize additional disrupted transmitter systems, highlighting many non-glutamate-related therapeutic possibilities for AD [142], for HD [143], and for ALS [144]. The common conclusion is that altering brain glutamate levels alone will not lead to clinical benefit.

So most reviews give a nod to the concept of glutamate excitotoxicity but avoid attributing disease progression to elevated and maintained glutamate release. More recently in reviews of the suspected biological pathways leading to AD, HD, and ALS, the term `excitotoxicity` is not even mentioned [145]. Glutamate levels are not elevated in HD, ALS, or AD over days, weeks, or months. This is not to say that glutamate neurotransmission may not be a therapeutic target in future. There is interest, for example, in modifying metabotropic gluRs in autism, epilepsy, and HD. But that is unrelated to hypothesized glutamate accumulation in the brain, a concept that is increasingly disregarded.

Additionally, there has recently been a call to reevaluate the confusing roles of gluRs in epilepsy [146]. One study, trying to argue that epilepsy was promoted by poststroke glutamate, found merely a slight increase in brain glutamate in patients sampled within 24 h of their stroke [147]. Given that time frame is when neurons are massively dying, the elevation is not surprising. Also, fully 93% of 102,008 poststroke patients did not develop seizures (Zou et al. 2015, cited in [147]), an expected consequence of elevated brain glutamate. Some animal studies have even demonstrated a decrease in glutamate levels post stroke [148].

Then there is TBI, another brain disorder where excess glutamate release has been held up as a cause and a target for treatment. Caution was advised in 1997 by Obrenovitch and Urenjak [122] who argued that the concept of glutamate excitotoxicity in TBI did not withstand scientific scrutiny. Cerebral microdialysis measurements of glutamate [149] showed that 43 of 165 patients displayed slight initial glutamate increases (> 20 μmol/L) which then normalized in 2 days; 74 were initially higher (~ 30 μmol/L) but normalized over 3 days. The remainder showed higher glutamate levels that randomly fluctuated. In other words, there was evidence for correlation between elevated glutamate and injury, but not causation. The fact that TBI can provoke release of the most common brain neurotransmitter is hardly surprising. Retrospectively, other studies showed that mortality was similar between patients who received excitatory amino acid inhibitors and those that received placebo [150]. In a recent review of prospective translational targets for new TBI therapies, excess glutamate was noted but ignored; SD was not even considered [151].

We conclude that the popular presumption in neuroscience that excess glutamate release drives neurodegeneration is based more on past dogma than on recent scientific evidence. There may be some clinical exceptions. Disorders associated with excess glutamate release are implicated as resulting from over-activation of the cysteine/glutamate antiporter. These include glioma-derived epileptic seizures and oligodendroglia death [82].

### Glutamate Excitotoxicity Theory Undermines the Importance of SD in Brain Injury

Recent articles reviewing brain ischemia generally default to excitotoxicity theory as the initial mechanism driving brain damage, for example [51, 97, 152–154]. This not because the hard evidence is in, but because there are so few competing theories. A recent and extensive review of global ischemia does not mention SD [155]. Many clinicians and researchers are unaware that recurring SD promotes acute brain damage following ischemia. Even then, SD may be cited simply as further promoting glutamate release and its related downstream pathways. These reviews ignore the litany of modifications to the glutamate hypothesis which themselves represent investigative and therapeutic dead-ends over the past 30 years. These include NMDA receptor blockade, intracellular calcium chelation, AMPA receptor inhibition, metabotropic gluR blockade, promotion of glutamate reuptake, upregulation of the gluR2 subunit, and inactivation of intracellular pathways downstream from NMDA receptors [50].

The dogma that glutamate excitotoxicity plays a major role in ischemic SD generation (as well as in post-SD injury) has had collateral effects. First, in some computer models, gluR-driven depolarization is often a supporting component driving SD, but with little experimental support from wet labs. Second and most importantly, SD is often considered merely an epiphenomenon of glutamate excitotoxicity. And in that respect SD joins focal stroke, global ischemia, HD, AD, ALS, and TBI as being consigned to a general repository for neurological disorders where glutamate excitotoxicity persists as a frustratingly vague process that somehow drives these disease entities.

### Therapeutic Hope for Combating Glutamate Excitotoxicity?

The drug candidate ketamine was discussed in “Ketamine and SD Inhibition” section. Another recently tested possible neuroprotective therapeutic is Nerinetide (NA-1). It is proposed to inhibit PSD-95, thereby preventing that protein from binding with the NMDA receptor. This then reduces the effects of Ca^2+^ influx which in turn [137] would activate lethal levels of NO [156]. Note that independently, preventing elevated NMDAR activation, or elevated intracellular Ca^2+^, or elevated NO has not protected from ischemic injury [50]. Furthermore, as explained above, it is unclear how NO should rise at all during severe ischemia as molecular oxygen is required for NO synthesis [136, 137]. But the NA-1 rationale is that the combined approach (downstream from SD) could be clinically efficacious [156]. While the drug did not help when administered with a clot buster, it had minor benefits on its own. There is also a related study, the Frontier Trial, wherein NA-1 was administered to stroke victims during hospital transport (https://nonoinc.ca/pipeline/). It may prove efficacious; if not, the commonly cited issue of too much variation among stroke patients will be proffered.

An additional recent therapeutic possibility is the introduction of glutamate grabbers into the general circulation based on the principle that excess glutamate in brain parenchyma can be promoted to cross the blood–brain barrier as glutamate is removed from the plasma [157]. The concept is noninvasive and can be administered over many days. The jury is still out.

Interestingly, Soria et al. [82] found that blockade of excitatory amino acid transporters or of vesicular glutamate release did not inhibit ischemia-gated currents or neuronal damage after OGD in slices but that pharmacological inhibition of the cystine/glutamate antiporter dramatically attenuated ischemia-gated currents and cell death after OGD. This antiporter needs to be further studied in relation to SD.

There may be some middle ground between SD generation and glutamate release, in that Na^+^/K^+^ ATPase has direct protein–protein interactions with the NMDA receptor [158]. The pump and the NMDA receptor form protein complexes on hippocampal dendrites; nanomolar concentrations of ouabain applied to single neurons causes an immediate and rapidly reversible reduction of the calcium response to NMDA receptor activation. So at least a subpopulation of pump complexes interacts directly with gluRs.

## Issues of Consensus Among the Authors

- SD evoked by the ischemia of focal stroke, cardiac arrest, subarachnoid hemorrhage, or TBI is itself a major contributor to the neuronal injury that follows. But the preeminence of glutamate excitotoxicity is continually reinforced by new review articles, primarily dealing with stroke, that do not question excitotoxicity theory, do not provide any new experimental support, and do not consider the important role of SD in ischemic brain damage. Meanwhile, recent reviews on other neurodegenerative diseases (AD, HD, ALS, TBI) have abandoned glutamate excitotoxicity as causal. We concur that more data are required to further support causality between SD and injury, and certainly between glutamate release and long-term brain injury.
- The molecular mechanisms leading to SD initiation are still unclear. Moreover, how SD regeneration drives propagation is poorly understood. Elevated levels of released extracellular K^+^ or of glutamate can help promote SD propagation, but neither are credible candidates as the biological initiator/perpetuator of SD. Both do play a role in K^+^-evoked and optogenetically-evoked SD where there is less metabolic stress to the gray matter. As a more viable explanation, there are hints that one or more small molecules may be released from gray matter during ischemia to initiate and then promote SD regeneration.

## Issues of Contention Among the Authors

- There is some contention regarding to what degree glutamate release during each SD event contributes to progressive injury. At one end of a spectrum is the scenario that recovery from each SD event alone elicits enough metabolic stress to expand neuronal injury independent of glutamate release. We all concur that the secondary injury cascade is important, but not that glutamate is the primary initiator. At the other end of the spectrum, some authors believe that gluR activation caused by SD-evoked glutamate release evoked is crucial in mediating expansion of the penumbra. In either scenario, the metabolic stress of SD is a major player in inducing acute neuronal injury.
- Some of the authors argue that the acceptance of glutamate excitotoxicity theory is stifling enquiry into other mechanisms of brain injury. And the sheer number of non-critical reviews reiterating the central role of glutamate release in brain injury is slowing the formulation of new ideas. Other authors counter that the central idea is essentially correct and that therapeutic advances in treating brain damage will eventually support the importance of released glutamate resulting from SD.
- That said, there continues to be few, if any, excitotoxicity-based clinical approaches to brain ischemia on the horizon. And the hope that glutamate excitotoxicity theory will provide insight to HD, AD, or ALS appears even more remote.

## Conclusions

The authors propose that understanding the etiology and molecular mechanisms underlying SDs will help clarify the complex field of ischemic brain injury. We suggest a reinterpretation of existing data in light of the clear prevalence of SD in patients suffering stroke, TBI, subarachnoid hemorrhage, or sudden cardiac arrest [1]. It is also important to note an emerging awareness that milder recurrent SD may generate the periodic bouts of disorientation associated with delirium [159] or with concussion [160]. Overall, a better understanding of the molecular events driving SD will enhance our ability to suppress SD events immediately post-injury, hopefully to benefit patient outcome.

## Abbreviations

SD: Spreading depolarization
OGD: Oxygen–glucose deprivation
gluR: Glutamate receptor
Kynurenate: 3-Anthraniloyl-l-alanine, broadly inhibits glutamate receptors
NMDA: *N*-methyl-d-aspartate (NMDA) receptor antagonists inhibit NMDA receptors
TEA: Tetraethylammonium
TTX: Tetrodotoxin
HD: Huntington’s disease
AD: Alzheimer’s disease
ALS: Amyotrophic lateral sclerosis
NO: Nitrous oxide
ROS: Reactive oxygen species
